# Diagnostic Value of Coronary Artery Calcium Score for Cardiovascular Disease in African Americans: The Jackson Heart Study

**DOI:** 10.9734/BJMMR/2016/21449

**Published:** 2015-09-21

**Authors:** Jung Hye Sung, Joseph Yeboah, Jae Eun Lee, Che L. Smith, James G. Terry, Mario Sims, Tandaw Samdarshi, Solomon Musani, Ervin Fox, Yaorong Ge, James G. Wilson, Herman A. Taylor, J. Jeffery Carr

**Affiliations:** 1 Department of Epidemiology and Biostatistics, Jackson State University, Jackson, Mississippi, USA; 2 Department of Internal Medicine/Cardiology, Wake Forest University Health Sciences, Winston Salem, North Carolina, USA; 3 Research Centers in Minority Institutions Translational Research Network Data Coordinating Center, Jackson, Mississippi, USA; 4 DARE Global Innovations, Washington DC, USA; 5 Department of Radiology and Vanderbilt Translational and Clinical Cardiovascular Research Center, Vanderbilt University, Nashville, Tennessee, USA; 6 University of Mississippi Medical Center, Jackson, Mississippi, USA; 7 Department of Software and Information Systems, The University of North Carolina at Charlotte, North Carolina, USA; 8 Cardiovascular Research Institute, Morehouse School of Medicine, Atlanta, Georgia, USA

**Keywords:** Coronary artery calcium, cardiovascular disease, African Americans

## Abstract

**Background:**

The role of coronary artery calcium (CAC) as a screening tool for cardiovascular disease (CVD) risk in African Americans (AAs) is unclear. We compared the diagnostic accuracy for CVD prevalence using the CAC score and the Framingham Risk Score (FRS) in an adult population of AAs.

**Methods:**

CAC was measured in 2944 participants AAs. Approximately 8% of this cohort had known CVD defined as prior myocardial infarction, stroke, percutaneous coronary intervention, coronary artery bypass grafting and peripheral artery disease. Logistic regression, receiver operating characteristic (ROC) and net reclassification index (NRI) analysis were used adjusting for age, gender, systolic blood pressure (SBP), total and high-density lipoprotein (HDL) cholesterol, smoking status, diabetes mellitus (DM), body mass index (BMI), blood pressure medication and statin use. Participants with prevalent clinical CVD and DM were classified as high FRS risk.

**Results:**

The mean age of participants was 60 years, 65% were females, 26% had DM, 50% were obese and 30% were current or former smokers. Prevalent CVD was associated with older age, higher SBP, lower HDL and total cholesterol, and higher CAC. The prevalence of CAC was 83% in participants with prevalent CVD and 45% in those without CVD. CAC was independently associated with prevalent CVD in our multivariable model [OR (95% CI): 1.22 (1.12–1.32), p< 0.0001]. In ROC analysis, CAC improved the diagnostic accuracy (c statistic) of the FRS from 0.617 to 0.757 (p < 0.0001) for prevalent CVD. Addition of CAC to FRS resulted in net reclassification improvement of 4% for subjects with known CVD and 28.5% in those without CVD.

**Conclusion:**

In AAs, CAC is independently associated with prevalent CVD and improves the diagnostic accuracy of FRS for prevalent CVD by 14%. Addition of CAC improves the NRI of those with prevalent CVD by 4% and the NRI of individuals without CVD by 28.5%. Determination of CAC may be useful in CVD risk stratification in AAs.

## 1. INTRODUCTION

Cardiovascular disease (CVD) is the leading cause of morbidity and mortality in the developed world [1]. Atherosclerosis is the underlying pathology for most cardiovascular diseases. Atherosclerosis progresses from early to advanced lesions, with subtypes of plaque that are relatively stable and others that are more high-risk for acute coronary syndromes [2,3]. Calcified plaques can indicate stable lesions as well as potentially lesions at higher risk, the so-called spotty calcifications [4]. Population-based studies have in general supported racial differences in the prevalence of calcified atherosclerotic plaques and suggest that Caucasians may have more calcified plaque than African Americans [5–7]. However, the predictive ability of calcified atherosclerotic plaques for hard events and cardiovascular death for black and whites have been comparable [8].

Coronary artery calcium score (CAC) is a quantitative measure of calcified atherosclerotic plaque that provides an estimate of the total atherosclerotic burden of the coronary circulation in an individual. CAC has been associated with cardiovascular risk factors and cardiovascular events, and has been shown to improve cardiovascular risk prediction over and above the Framingham Risk Score [9–12]. The assessment of CAC was given a class II indication in the recent American Heart Association/American College of Cardiology guideline for cardiovascular risk assessment in asymptomatic individuals [13]. The lower prevalence of calcified plaques in African Americans raises concerns that CAC in African Americans may not have the same implications or inform clinical decision making to the same degree as in Caucasians who have the highest prevalence. The diagnostic accuracy of CAC for cardiovascular events and the improvement afforded by the addition of CAC to traditional CVD risk factors and the FRS in African Americans is therefore unclear.

To address some of the limitations in current data on the association of CAC and CVD in African Americans, we assessed the diagnostic accuracy of CAC and the improvement afforded by CAC over the Framingham Risk Score (FRS) for prevalent clinical cardiovascular disease in African Americans who were part of the Jackson Heart Study, an NIH/NHLBI sponsored study based in Jackson Mississippi.

## 2. METHODS

### 2.1 Study Participants

The Jackson Heart Study (JHS) is a single-site, prospective cohort study of the risk factors and causes of cardiovascular disease in adult African Americans. A probability sample of 5301 African Americans, 21 to 84 years of age, residing in the three counties surrounding Jackson, MS, were recruited and examined at baseline (2000–2004) by trained and certified technicians according to standardized protocols. Clinic visits and interviews occurred approximately every three years. Annual follow-up interviews and cohort surveillance are ongoing. Details of the study design have been previously published [14,15].

For all participants, the clinic visit included physical examination, anthropometry, survey of medical history and of cardiovascular risk factors, and collection of blood and urine for biological variables. We calculated body mass index (BMI; kg/m^2^) as weight in kilograms divided by height in meters squared. Obesity was defined as BMI ≥30 kg/m^2^ and abdominal obesity as a waist circumference ≥88 cm in women and ≥102 cm in men. Hypertension was defined as systolic blood pressure ≥140 mm Hg, diastolic blood pressure ≥90 mm Hg, or use of antihypertensive therapy. Lipid profile was measured under standard laboratory conditions (16). Diabetes mellitus (DM) was defined as fasting plasma glucose ≥126 mg/dl or use of insulin or oral hypoglycemic medications. Smoking status was defined as current smoking versus former and never smoking (collapsed). Alcohol drinking was defined as regular drinking in the past 12 months (yes versus no). A physical activity score was composed with a Baecke-derived questionnaire and used as a continuous variable.

For the current study, 2,459 participants were excluded thus: participants without information on CAC from the CT scan Exam and those without information on the prevalent CVD. After these exclusions, 2,842 participants were eligible for our study.

### 2.2 Coronary Artery Calcium Score Measurement

Computed tomography (CT) imaging of the torso were obtained by multi-detector CT (GE Healthcare Lightspeed 16 Pro, Wakeshau, Wisconsin) during Exam 2 (dates first to last CT) at the Jackson Medical Mall. The participants were scanned in the supine position with a three sample calcium calibration QCT Phantom (Image Analysis, Columbia, KY) posterior to the spine. The phantom is made from tissue equivalent plastic and contains rods of known concentration of calcium hydroxyapatite (0, 75 and 150 mg/cc). Technical settings include: prospective ECG gating at 75% of the R-R interval, 120 KVp, 2.5 mm slice thickness, 35 cm display field of view, gantry speed was 0.40 seconds and a segmented reconstruction (aka half-scan) resulting in an effective temporal resolution of 0.24 seconds. Tube current was 400 mA and increased by 25% for participants weighing ≥220 lbs (100 Kg) to compensate for body size and maintain a more constant signal-to-noise ratio across participants. The entire CT protocol for the Jackson Heart Study included series for imaging the heart, liver and abdomen and for the total exam participants received a one-time exposure of less than 6 mSv. Scans were read centrally at the Wake Forest University School of Medicine. Coronary artery calcium scoring was performed using previously described methods for large epidemiologic trials [17]. Images were viewed and scored using a TeraRecon Aquarius Workstation (TeraRecon, Inc., San Mateo, CA).

### 2.3 Prevalent Cardiovascular Disease

Upon completion of the JHS Exam 2 clinic visit and associated surveys, prevalent CVD was determined to be about 8% in the cohort. Prevalent clinical CVD in the JHS is defined as prior myocardial infarction, stroke, percutaneous coronary intervention, coronary artery bypass grafting and peripheral artery disease as documented during the clinic visit.

### 2.4 Statistical Analysis

Demographic characteristics of the cohort are presented as means standard deviations for continuous variables and frequencies for categorical variables. CAC was transformed [In (CAC +1)] and used in our models. The FRS of each participant was calculated for each participant using the approach by D’Agostino et al. [18]. Individuals with prevalent clinical CVD or diabetes mellitus were classified as “high” Framingham risk and were all given a score of greater than 20%. Logistic regression analyses were used to assess the association between CAC and prevalent clinical CVD in the univariate and multivariable model adjusting for confounders such as age, gender, systolic blood pressure, total and HDL cholesterol, BMI, cigarette smoking status, diabetes mellitus, blood pressure medication and statin use.

Receiver operator curve analysis was done using the calculated FRS alone and with CAC to determine the improvement of the area under the curve (AUC) afforded by the addition of CAC to the FRS for the diagnosis of prevalent clinical CVD. The cohort was subsequently divided into three CV risk categories based on the FRS.; low (0–10%), intermediate (10–20%) and high (>20%). Predicted probabilities for prevalent clinical CVD were calculated in logistic regression models for each participant and were used to create revised FRS categories [19]. The revised low, intermediate and high FRS categories were (0 – 10.1%), (10.1 – 19.6%) and (>19.6%) respectively specific for the participants of the JHS. CAC was then added to FRS in the logistic regression models and based on the new predicted probabilities; participants were reclassified as low, intermediate and high risk. Net reclassification improvement (NRI) was then calculated separately using the formula for participants with and without prevalent clinical CVD at baseline. The combined NRI was then calculated by adding the NRI of participants with prevalent clinical CVD and those without prior CVD. All statistical analyses were performed with the use of SAS version 9.2 (SAS Institute, Cary, NC).

## 3. RESULTS

A total of 2,842 participants, 224(8%) with prevalent clinical CVD, had complete data and were included in this analyses. As expected, participants with prevalent clinical CVD were older, had higher blood pressure, greater CAC and were more likely to be males, cigarette smokers and have diabetes mellitus (Table 1). The mean CAC score in participants with and without know prevalent clinical CVD was 576.9 and 141.2 Agatston respectively.

**Fig. 1:**
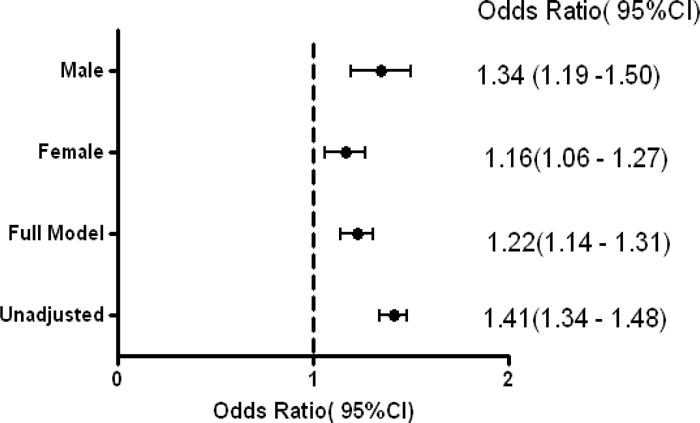
Association between coronary calcium score and prevalent cardiovascular disease in the unadjusted and fully adjusted models and in full adjusted models stratified by gender in the Jackson Heart Study

**Fig. 2:**
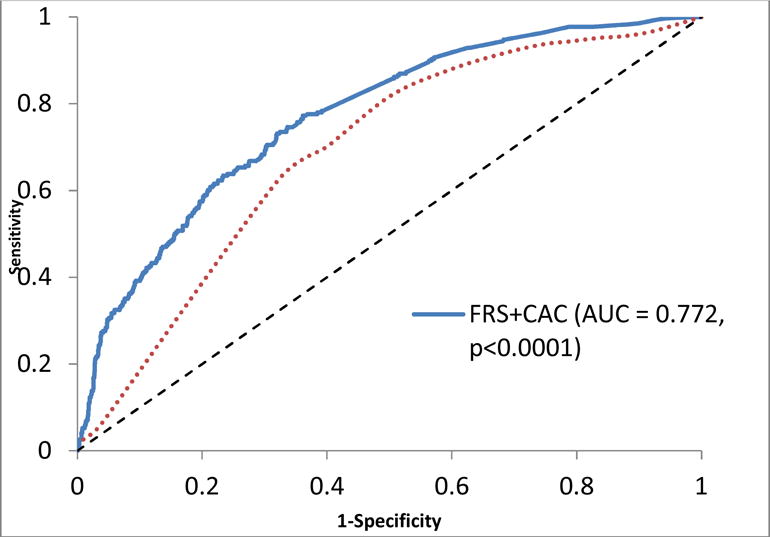
Improvement in diagnostic accuracy afforded by the addition of coronary Artery calcium score (CAC) to the Framingham Risk Score (FRS) for prevalent cardiovascular disease in African Americans

**Fig. 3:**
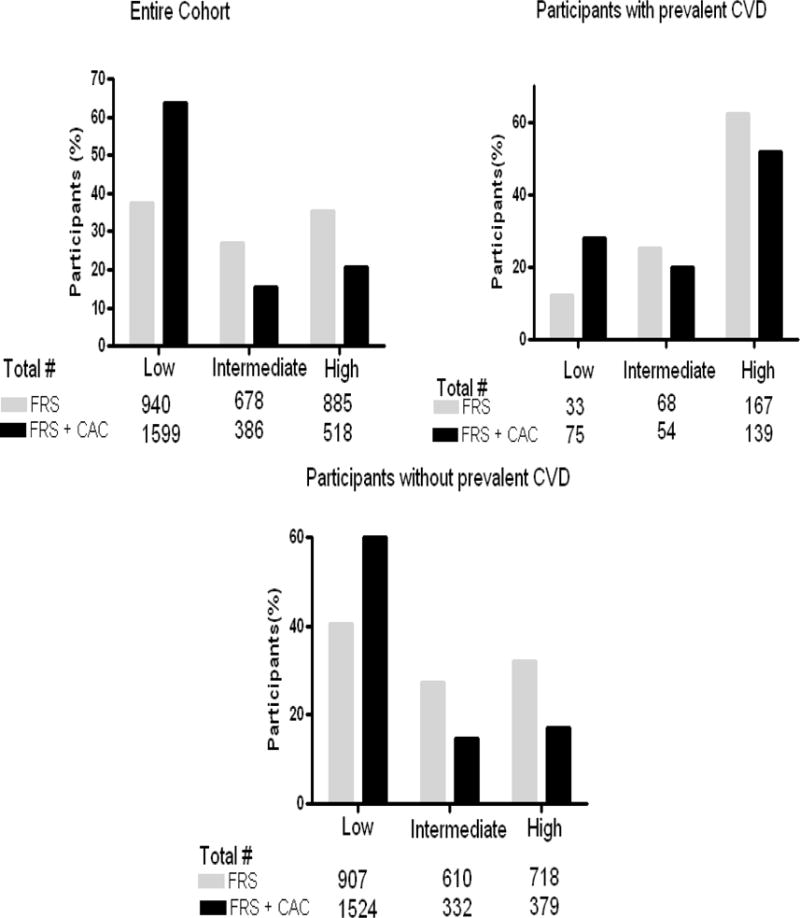
Reclassification of risk associated with the addition of coronary calcium score (CAC) to the Framingham Risk Score (FRS) for prevalent cardiovascular disease (CVD) in the Jackson Heart Study

CAC was associated with prevalent clinical CVD in the univariate and multivariable logistic regression models [OR (95%CI): 1.41(1.33 – 1.48, p<0.0001 and 1.22 (1.12–1.32), p< 0.0001 respectively]. In stratified multivariable analyses, CAC was associated with prevalent clinical CVD in both males and females [OR (95%CI): 1.29(1.12 – 1.48, p=0.0003 and 1.17(1.06–1.31), p=0.003 respectively] (Fig. 1). As shown in Fig. 2, the addition of CAC to the FRS improved the diagnostic accuracy (c statistics) from 0.617 to 0.757 (p < 0.0001) for prevalent clinical CVD. Using the FRS, 30% of the cohorts were classified as high risk, 38.5% were classified as intermediate and 31.5 were classified as low risk. In all, 51% of those with prevalent CVD were classified as high risk by the FRS. Moreover, 46% of those with known CVD and 57% of those without prevalent disease were reclassified into a different risk category by the addition of CAC to the FRS. The addition of CAC improved the diagnostic accuracy of the FRS by 4% in participants with known CVD and 28.5% in participants without known CVD. The overall NRI was 0.325 or 32.5% (Fig. 3).

## 4. DISCUSSION

The goal of this study was to assess the diagnostic accuracy of the Framingham Risk Score (FRS) and explore the improvement in the diagnostic accuracy by the addition of CAC in African Americans (AAs); a race/ethnic group with high cardiovascular risk, lower prevalence of CAC compared to white population, and in whom the FRS is suboptimal. The present study showed that blinded to CVD status, the FRS classifies 51% of AA with known CVD appropriately as high risk and even though CAC significantly improve the c statistics of the FRS, the addition of CAC only improved the reclassification of FRS by 4% in participants with known CVD. However in the majority of JHS participants, those without prevalent CVD, the CAC was more informative resulting in the number of individuals classified as “intermediate risk” decreasing from 40% to ~ 16%. The majority of these individuals moved from intermediate to low risk groups with the low risk group increasing from 33% to 60%.

Current data supports race/ethnic differences in the prevalence and amount of coronary artery calcification [5–7]. Non-Hispanic whites have been shown to have higher prevalence and severity of coronary calcification compared with other race/ethnicities including AA [20]. However, African Americans have been shown to have higher cardiovascular risk factor burden and cardiovascular event rates compared with non-Hispanic whites. In the present study, 83% of participants with prevalent CVD had CAC compared with 45% of participants without prevalent CVD with significant differences in CAC burden. Thus AA men and women with CVD have a very high prevalence of CAC and high burden of CAC.

Several studies have evaluated the improvement in the diagnostic/prognostic accuracy of the FRS using CAC and have shown consistent positive results [9–13]. Based on these studies the NCEP/ATP III guidelines, a modification of the FRS, has recommended CAC screening in all race/ethnicities [14]. However, minorities are underrepresented in these studies [10–12] and despite attempts at subgroup analyses, the results have not been conclusive. In the present study, the addition of CAC to the FRS only improved the NRI by 4% for the diagnosis of clinical CVD in this cohort of African Americans. Even though CAC was used in our models for diagnosis of prevalent CVD while most other studies have used it for prediction of future CVD events, the improvement in NRI for the diagnosis of CVD was still significantly lower compared with improvement in NRI afforded by the addition of CAC to FRS in prediction models in mostly non-Hispanic white populations. Sufficiently powered studies evaluating the improvement in prognostic accuracy of the FRS by CAC for incident coronary/cardiovascular events in African Americans and other minorities groups are needed. Ongoing surveillance of the JHS will allow a future report to describe the prospective ability of CAC to predict CVD events.

Our study has the following limitations. First, prevalent CVD is self-reported and therefore there may be an inherent bias due to under or over reporting. This is cross sectional analysis of an ongoing observational study and even though most covariates were accounted for in our models, residual confounding may have influence our findings. Third, despite diagnostic value of the CAC for cardiovascular disease which was proved by this study, ethical issue still remained in clinical use of CAC as diagnostic tool due to increased risk of radiation. Therefore, there may be a need for an alternative diagnosis that could be reached with other non-invasive and safer techniques. Finally, our study is in AA in Jackson, MS and may not apply to other race/ethnic groups. Further research investigating racial differences between CAC and CVD should be performed.

## 5. CONCLUSION

In AA, CAC is independently associated with prevalent clinical cardiovascular disease. Moreover, CAC significantly improves the diagnostic accuracy of the FRS for prevalent clinical CVD in ROC analysis and net reclassification index analysis. Interestingly the improvement in discrimination afforded by the addition of CAC to FRS appears to be greater in the low risk compared with high risk participants.

## Figures and Tables

**Table 1:** Demographics and risk factors by prevalent clinical cardiovascular disease status

Variables	Prevalent CVD (N=224) (Mean±SD)	No CVD (N=2618) (Mean±SD)	P value
Age (years)	66.9±9.1	58.9±10.9	<0.0001
Male gender (%)	95 (42.4)	906 (34.6)	0.012
BMI (Kg/m^2^)	31.4±5.8	31.7±6.5	0.39
**Cigarette smoking (%)**			<0.0001
Never	113 (50.4)	1873 (71.5)	
Former	68 (30.3)	481 (18.4)	
Current	43 (19.3)	264 (10.1)	
**Cholesterol (mg/dl)**			
Total	180.6±40.4	198.2 40.3	<0.0001
HDL	51.3±12.7	54.8±15.4	<0.0001
LDL	106.7±36.5	122.9±36.5	<0.0001
Triglycerides	118.2±90.3	104.7±91.9	0.07
Diabetes mellitus (%)	89 (39.7)	542 (20.7)	<0.0001
**Blood pressure (mmHg)**			
Systolic	130.8±22.0	126.7±18.2	0.009
Diastolic	73.7±10.6	77.4±10.5	<0.0001
BP medication use (%)	184 (82.1)	1516 (57.9)	<0.001
Statins use (%)	78 (34.8)	259 (9.9)	<0.0001
Calcium score (Agatston)*	576.9±903.1	141.2±479.2	<0.0001

## ABBREVIATIONS

CAC: Coronary Artery Calcium
CVD: Cardiovascular Disease
AAs: African Americans
FRS: Framingham Risk Score
ROC: Receiver Operating Characteristic
NRI: Net Reclassification Index
SBP: Systolic Blood Pressure
HDL: High-density Lipoprotein cholesterol
DM: Diabetes Mellitus
BMI: Body Mass Index
OR: Odds Ratio
CI: Confidence Interval
JHS: Jackson Heart Study
NIH/NHLBI: National Institutes of Health/National Heart, Lung, and Blood Institute
CT: Computed Tomography
ECG: Electrocardiogram
AOC: Area under the Curve
NCEP/ATP III: National Cholesterol Education Program/Adult Treatment Panel
